# Accumulation of Fungal Pathogens Infecting the Invasive Spotted Lanternfly, *Lycorma delicatula*

**DOI:** 10.3390/insects14120912

**Published:** 2023-11-27

**Authors:** Ann E. Hajek, Thomas A. Everest, Eric H. Clifton

**Affiliations:** 1 Department of Entomology, Cornell University, Ithaca, NY 14853, USA; te97@cornell.edu (T.A.E.); eclifton88@gmail.com (E.H.C.); 2 Research & Development, BioWorks Inc., Victor, NY 14564, USA

**Keywords:** entomopathogenic fungi, invasive, opportunistic fungal pathogens, planthopper, biological control

## Abstract

**Simple Summary:**

Populations of the invasive spotted lanternfly in the eastern United States threaten vineyards and pose a public nuisance. Methods for control are being investigated, with an interest in mortality caused by insect-specific pathogens and naturally occurring predators. Two species of insect-pathogenic fungi caused extensive mortality in spotted lanternfly populations in fall 2018, and two additional fungal pathogens have been reported. Our extensive surveys, which emphasized a year with a summer drought, document that while *Beauveria bassiana* was most abundant, fifteen additional fungal pathogens killed spotted lanternflies. Although levels of infection for many pathogen species were low, infection was greatest in adults sampled in September and October, when spotted lanternfly reproduction occurs. Thus, we report an increased diversity of naturally occurring generalist fungal pathogens attacking this relatively new invasive planthopper.

**Abstract:**

In the eastern United States, populations of the invasive spotted lanternfly, *Lycorma delicatula*, are abundant and spreading. Four species of naturally occurring entomopathogenic fungi have previously been reported as infecting these planthoppers, with two of these causing epizootics. Nymphal- and adult-stage lanternflies in Pennsylvania and New York were surveyed for entomopathogenic fungal infections from October 2021 to November 2023, and assays were conducted to confirm the pathogenicity of species that were potentially pathogenic. *Beauveria bassiana* was the most abundant pathogen, but we report an additional 15 previously unreported species of entomopathogenic fungi infecting spotted lanternflies, all in the order Hypocreales (Ascomycota). The next most common pathogens were *Fusarium fujikuroi* and *Sarocladium strictum*. While infection prevalence by species was often low, probably impacted to some extent by the summer drought in 2022, together these pathogens caused a total of 6.7% mortality. A significant trend was evident over time within a season, with low levels of infection among nymphs and higher infection levels in mid- and late-stage adults, the stages when mating and oviposition occur.

## 1. Introduction

The success of invasive species in their introduced ranges is often attributed to escape from natural enemies that provide control in native regions [1]. However, over time, natural enemies can adapt to using an invasive species through host shifts, evolutionary changes in resident enemy populations, or introductions of co-evolved natural enemies [2,3,4]. In the case of pathogens, the accumulation of pathogens attacking invasives has been hypothesized to potentially (1) lead to population decline of invasives or (2) create increases in pathogen populations that could spill over to impact co-occurring native host species [4]. With no saturation in numbers of invasive insect species predicted over time [5], the potential accumulation of entomopathogens attacking invasives, relative to both their biodiversity and their impact, requires investigation.

Entomopathogenic fungi are often found infecting invasive insect populations, especially when those invasive hosts are abundant [6]. In simplified ecosystems, epizootics in populations of one host species can occur, predominantly driven by one or two fungal species, and these are frequently well-known species in just a few genera, including species of *Beauveria*, *Metarhizium*, *Akanthomyces* (previously *Lecanicillium*), and *Cordyceps* [6]. However, there are more than 1000 species of entomopathogenic fungi in many different genera [7,8]. There are numerous reports of multiple entomopathogenic fungi infecting invasive insect populations [9,10,11,12,13,14,15,16,17,18], with many of these entomopathogens belonging to the order Hypocreales and considered native to the region where the pest is invading. These hypocrealean entomopathogens infecting invasives range from well-known pathogens, such as *Beauveria bassiana*, to opportunists requiring specific conditions (e.g., impaired host immunity) to infect insects [19].

The spotted lanternfly, *Lycorma delicatula*, is originally from Asia but was first discovered in Berks County, Pennsylvania, in 2014 and has since spread into 15 additional eastern US states [20]. This univoltine species feeds on woody plants, with nymphs feeding on leaf midribs, petioles, and twigs and adults feeding through bark. Once an area has been invaded, this planthopper increases in abundance, often in areas where its preferred Asian host tree, tree of heaven (*Ailanthus altissima*), occurs. Another preferred plant for *L. delicatula* is grape (*Vitis* spp.), and feeding has caused decreased yield in vineyards, requiring new control tactics [21]. Hemiptera, including planthoppers, are particularly susceptible to entomopathogenic fungi [7,22], and a species known worldwide, *B. bassiana*, has been reported to infect *L. delicatula* where it is native, in China [23].

In 2018, epizootics caused by entomopathogenic fungi were documented in abundant populations of *L. delicatula* in southeastern Pennsylvania [24]. One fungus causing these epizootics was the well-known insect pathogen *B. bassiana*, while the second was the lesser-known *Batkoa major*. *Batkoa major* is a poorly known obligate insect pathogen [25], while *B. bassiana* is a significant pathogen of insects, known to infect >700 insect species, but which can also live as a saprophyte and plant endophyte [26]. Late in December 2018 and in November 2020, two new species of entomopathogenic fungi were discovered infecting *L. delicatula*: *Metarhizium pemphigi* and *Ophiocordyceps delicatula* [27]. These discoveries were not previously known from North America and not previously described, respectively. Thus, by 2020, four entomopathogenic fungi were known to naturally infect *L. delicatula* in the US. In 2021, three more species were isolated from *L. delicatula* adults (see below), and the need for the present study became apparent. The goal of this study was to evaluate the diversity and abundance of entomopathogenic fungi naturally infecting *L. delicatula* of different stages in invasive infestations in the northeastern US. Fungal species were isolated and identified using molecular methods, and tests were conducted to confirm pathogenicity when needed.

## 2. Materials and Methods

### 2.1. Sample Collection

Throughout our studies, because *L. delicatula* is not considered well established in the area around Cornell University, all work with living *L. delicatula* was conducted under permit (USDA APHIS permit # P526P-21-02895) within the Cornell arthropod quarantine building, the Sarkaria Arthropod Research Laboratory (SARL). Any living *L. delicatula* collected at field sites were transported in triple containment to SARL for our studies.

This study was initiated with fungal entomopathogens isolated from 3 *L. delicatula* collected from two Pennsylvania sites in October and early November 2021. All three were adults; one was collected living and died while being reared on *A. altissima* (see below), and the other two were cadavers of recently-killed adults. In 2022, 4 field sites in eastern Pennsylvania were chosen where *L. delicatula* and *A. altissima* were well established in forested areas. Between 8 June and 2 November, 15 trips were made to these sites for collections (Table 1). Two additional trips were made to Angora Fruit Farm and Sinking Spring on 29 September and 12 October. On each trip, approximately 30 *L. delicatula*, representing the stages present at that site at that time, were collected. The *L. delicatula* collected alive were reared on potted *A. altissima* plants in netting cages in a growth chamber at 22.5 °C (days) and 15 °C (nights), 14:10 (L:D), and 65% relative humidity, as described in Clifton and Hajek [28]. Reared *L. delicatula* were monitored daily for 14 d after collection, and any *L. delicatula* that died were removed daily from cages. To promote fungal outgrowth, cadavers of first–third-instar nymphs were moved into individual wells in 24-well plates (Falcon 353047). Each of these plates was maintained in a tightly closed plastic container (17 × 17 × 5 cm), with wet paper towels to maintain high humidity. Cadavers of fourth-instar nymphs and adults were placed individually in 29 mL clear, tightly-lidded plastic cups containing 6 mL of 1.5% water agar.

During each collection trip in 2022, the ground was also searched for cadavers of *L. delicatula* that had died very recently, with no evidence of scavenging. Searching for cadavers occurred for either 15 min or until 30 cadavers had been collected. Cadavers were placed individually in water agar cups and were treated similarly to cadavers of reared *L. delicatula* that died in the quarantine laboratory. All cadavers from both rearing and cadaver collections in the field were kept under humid conditions at 23 °C for 1–4 weeks. In 2022, a total of 2177 living and 373 dead *L. delicatula* were collected. Among the live *L. delicatula*, 1097 (50.4%) died within 14 d while being reared.

In 2023, *L. delicatula* populations at Glen Run and Angora Fruit Farm were sampled on 20 July, and at each site, 20–38 live *L. delicatula* were collected and reared in the quarantine laboratory. An infested site in Owego, New York (Table 1), was also visited on 7 and 12 July and on 9 August, and 43–55 living *L. delicatula* were reared from each collection date. In 2023, the same procedures were employed, monitoring insects for 14 d and maintaining any resulting cadavers under high humidity. However, in 2023, immediately after discovery, cadavers were surface-sterilized with the following methods: rinsing in 0.75% sodium hypochlorite for 30 s, followed by rinsing with deionized water for 30 s before incubating at high humidity to promote fungal outgrowth.

### 2.2. Fungal Isolation and Identification

Fungi were isolated from outgrowth on each cadaver and grown on potato dextrose agar (PDA) with 0.3 g/L streptomycin at 23 °C for several weeks before being moved to 4 °C. Cultures were then grouped into morphotypes based on physical characteristics. Representatives of each morphotype were selected for sequencing and were then subcultured and grown at 23–25 °C until sampling for DNA extraction.

DNA was extracted from young cultures using the DNeasy Plant Mini Kit (Qiagen, Germantown, MD, USA). Fungal mycelium was added to AP1 buffer with 0.5 gm of 0.7 mm zirconia beads (BioSpec, Bartlesville, Oklahoma, USA) and beat for 1 min at 4.8 krpm. After bead beating, samples were incubated at 65 °C for 10 min with inversion every 3 min; all other steps were carried out following the manufacturer’s instructions. PCR was conducted on up to 5 loci for each isolate; primers used are listed in Table 2. The nuclear ribosomal DNA internal transcribed spacer region (ITS1-5.8S-ITS2 = ITS) was amplified for exemplar isolates of each species (Table 3) and most of the additional samples requiring sequencing (see Table S1) following the protocol of White et al. [29]. The nuclear ribosomal DNA large subunit (LSU) was amplified for some Cordycipitaceae samples following the protocol in Vilgalys and Hester [30]. The protein-encoding gene RNA polymerase II largest subunit (RPB1) was amplified for some samples of Cordycipitaceae following the protocol of Castlebury et al. [31] but with an initial denaturation of 2 min at 94 °C and annealing temperatures of 52 °C for *Flavocillium bifurcatum* and 45 °C for other Cordycipitaceae. The protein-encoding gene RNA polymerase II second-largest subunit (RPB2) was amplified for *Samsoniella* sp., *Fusarium fujikuroi*, and *Fusarium graminearum* (primer pair RPB2-5F2 and RPB2-7cR) following protocols of Sung et al. [32] but with an initial denaturation of 2 min at 94 °C, an annealing temperature of 57 °C [33], and a final extension of 5 min at 72 °C. For *Akanthomyces muscarius*, *F. bifurcatum*, *F. fujikuroi*, *F. graminearum*, and *Samsoniella* sp., RPB2 was amplified following Liu et al. [34] (primer pair RPB2-5F and RPB2-7cR). The protein-encoding gene translation elongation factor 1-α (TEF1-α) was amplified for most samples, following protocols of Rehner and Buckley [35] for Cordycipitaceae (primer pair 983F and 2218R) and Karlsson et al. [36] for *Fusarium* and *Clonostachys* spp. (primer pair EF-1 and EF-2). The protein-encoding gene ß-tubulin (TUB2) was amplified for *Colletotrichum fioriniae*, *Cordyceps javanica*, and *Samsoniella* sp. following the protocol in Weir et al. [37].

For specific identification, genera and species with records as insect pathogens in the literature were emphasized, and genera that are often considered common saprophytes, e.g., *Cladosporium*, *Mucor*, and *Penicillium* [15], were not evaluated further. *Beauveria bassiana* samples were excluded from molecular analyses because these cultures could be identified morphologically, and *B. bassiana* is the only *Beauveria* species that has been isolated from *L. delicatula* during extensive sampling in eastern Pennsylvania [45]. For each species, an exemplar isolate was chosen, sequences were deposited in GenBank, and the culture was deposited in the ARSEF culture collection (Table 4). When there were multiple samples of one species, usually these were also sequenced (Table S1), except in cases where the morphotype of the cultures were very distinctive (i.e., *Sarocladium strictum*). All samples were sequenced by the Biotechnology Resource Center, Cornell University, and resulting sequences were aligned and trimmed with Geneious Prime (2023.0.4). For identification, *Fusarium* samples were compared with sequences in the FUSARIOID-ID database [72], and all other samples were compared with sequences in the NCBI GenBank database. In cases where a clear species identity was not apparent after comparing sequences of multiple loci to the relevant database (i.e., *A. muscarius* and *F. bifurcatum*), morphology of the isolate was also evaluated for identification.

### 2.3. Testing Pathogenicity

In 2021 and 2022, cadavers of *L. delicatula* from rearing field collections were not surface-sterilized after death, creating the possibility that fungal isolates from cadavers were saprophytic and not pathogenic. Therefore, tests were conducted to confirm pathogenicity to *L. delicatula*, using the sequenced exemplar isolate for each fungal species. In 2023, a pathogenicity test was also conducted with *C. fioriniae*, although the cadaver had been surface-sterilized. Based on field and laboratory studies with *B. bassiana* [24,28], tests of pathogenicity were not necessary, and based on laboratory bioassays with *C. javanica* [28], complete pathogenicity testing was unnecessary (i.e., controls were not included during pathogenicity tests and numbers of *L. delicatula* tested were decreased).

To test pathogenicity, exemplar isolates were grown on PDA or SDA (Sabouraud dextrose agar) (Table S2) until adequate sporulation occurred. For some *Fusarium* isolates, growth under black lights without parafilm was necessary to promote conidial production (K. Myers and G. Bergstrom pers. comm.). Conidia were removed from plates by adding 6 mL of sterile 0.05% Tween per plate, disrupting the cultures with a glass cell spreader, and pouring the resulting suspension through cheesecloth to remove hyphae and agar. Most pathogenicity tests were conducted with 1 × 10^7^ conidia/mL, although the initial tests with isolates from 2021 collections used 3.5 × 10^7^ conidia/mL for *Fusarium falsibabinda*, 7.4 × 10^6^ for *Cordyceps cateniannulata*, and 6.7 × 10^7^ for *A. muscarius*.

*Lycorma delicatula* used in pathogenicity tests were collected in the field and caged on plants in the quarantine growth chamber for 1–12 d prior to pathogenicity tests. Because *L. delicatula* is univoltine and year-round, non-diapausing colonies were not available, and we used the life stages present in the field at the times that sporulating cultures were ready (Table S2). For inoculations, 20 *L. delicatula* nymphs or adults (as an exception, 6 were used for *C. javanica*) were cold-anesthetized at 4 °C for 2 min, placed in groups of 1–3 in centrifuge tubes containing a conidial suspension, and shaken by hand for 10 s. Insects were then briefly placed on a paper towel so that excess water would drain off and were then reared on *A. altissima* in cages for 14 d. For the first 3 d after inoculation, cages were enclosed in clear plastic bags with wet paper towels to maintain high humidity. For 14 d after inoculation, cages were monitored daily, and cadavers were removed and maintained individually under high humidity. For controls, 20 *L. delicatula* of the same stage being challenged with fungi were treated the same way but were initially shaken in groups in 0.05% Tween. For pathogenicity tests with the 3 species first collected in 2021, controls were not included, but additional *L. delicatula* caged nearby were observed, and mortality due to these fungi (*A. muscarius*, *C. cateniannulata*, and *F. falsibabinda*) was not observed. For pathogenicity tests with 2021 samples, subsequent fungal outgrowth from dead insects was examined to confirm fungal identity, while for tests with samples from 2022–23, fungi were reisolated from cadavers and cultures were examined to confirm identity.

### 2.4. Photographing Fungal Forms

Photos of fungal outgrowth from *L. delicatula* cadavers, conidia, and obverse and reverse sides of cultures were taken for exemplar isolates known to be pathogens. After the death of an infected insect, cadavers were maintained under high humidity. For most species, approximately 1 wk after death, fungal outgrowth was adequate, and the cadaver was stored at 4 °C until a photo was taken under a dissecting microscope at 7.5× magnification for adults and 10–15× for nymphs. Conidia were harvested and photos were taken at 400× under phase contrast, except in the case of *F. graminearum*, for which phase contrast was not used. Photos of cultures were all taken on PDA in 100 mm petri dishes. Photos of *B. bassiana* are not included because the appearance of this species is already well-documented [24,45].

### 2.5. Analyses

Comparisons of infection prevalence were conducted using data from 2022, when consistent, extensive sampling occurred across the season. To evaluate levels of infection by stage across the 2022 season, first–third instar nymphs were merged, 4th-instar nymphs were considered separately, and adults were grouped into three stages. Stage A1, extending from eclosion to 7 September, is spent feeding, aggregating, and beginning to disperse [73]. Stage A2, extending from 8 September to 5 October, is when courtship and mating occur and oviposition begins, and Stage A3, extending from 6 October to 2 November, includes oviposition as the major behavior, with limited courtship and mating. For analyses, aside from *B. bassiana*, insect pathogens are grouped as ‘opportunists’ [19], based on their lower abundance in this study or the fact that most were not generally considered major fungal entomopathogens [6].

The percentage of *L. delicatula* infected was calculated for each instar/stage by the number infected insects by all insect pathogens identified in this study divided by the total number of that instar/stage collected, merging data across sample dates. Frequencies by instar/stage were compared using chi-squared tests with a Bonferroni correction. The numbers of males versus females that died from *B. bassiana* or opportunistic pathogens (all other pathogens) were compared using chi-squared tests. Analyses were conducted using SAS (Version 9.4, SAS Institute, Inc., Cary, NC, USA).

## 3. Results

### 3.1. Pathogen Biodiversity

**Figure 1:**
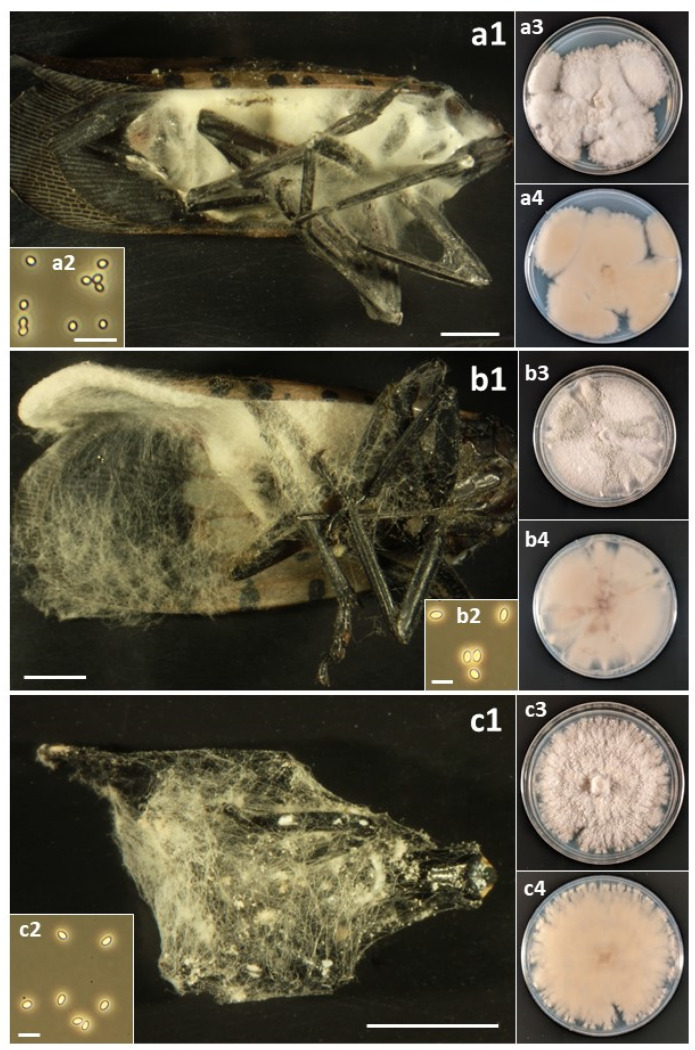
(a) *Akanthomyces muscarius*: (**a1**) Infected *L. delicatula* adult. (**a2**) Conidia. (**a3**) Colony on potato dextrose agar (PDA) at 23 d, obverse. (**a4**) Colony on PDA at 24 d, reverse. (b) *Clonostachys eriocamporesii*: (**b1**) Infected *L. delicatula* adult. (**b2**) Conidia. (**b3**) Colony on PDA at 36 d, obverse. (**b4**) Colony on PDA at 36 d, reverse. (c) *Clonostachys rosea*: (**c1**) Infected *L. delicatula* third-instar nymph. (**c2**) Conidia. (**c3**) Colony on PDA at 24 d, obverse. (**c4**) Colony on PDA at 24 d, reverse. Cadaver scale bars: 3 mm. Conidial scale bars: 10 µm.

**Figure 2:**
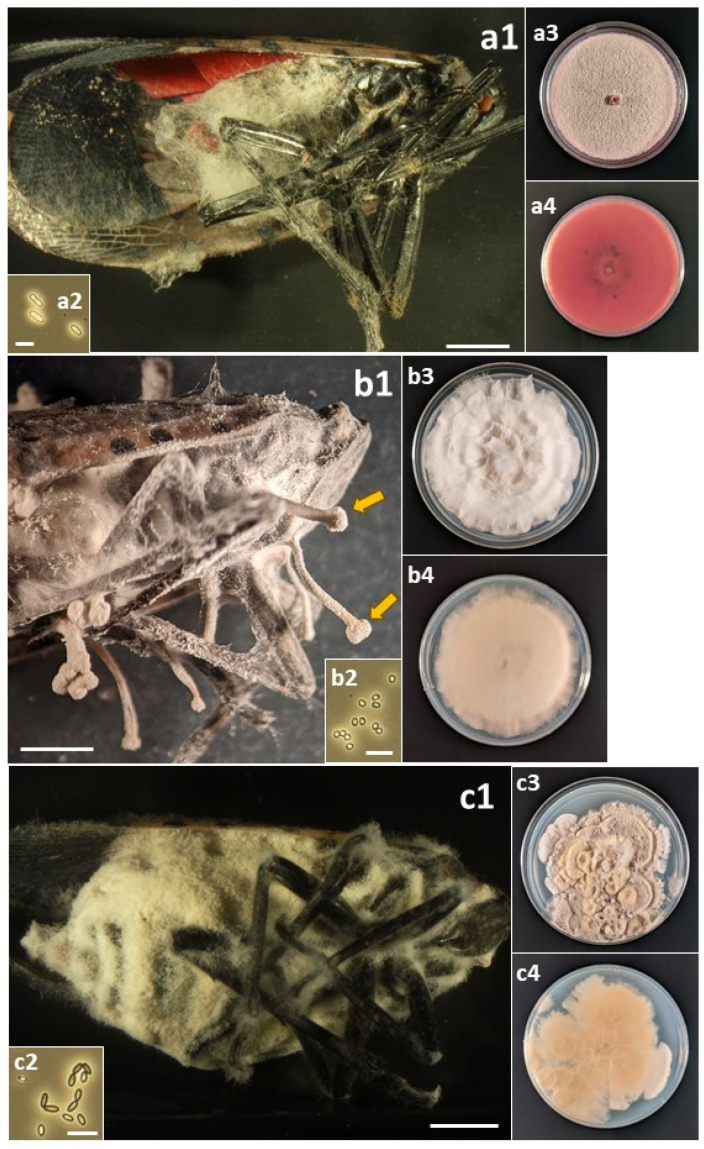
(a) *Colletotrichum fioriniae*: (**a1**) Infected *L. delicatula* adult. (**a2**) Conidia. (**a3**) Colony on PDA at 12 d, obverse. (**a4**) Colony on PDA at 12 d, reverse. (b) *Cordyceps cateniannulata*: (**b1**) Infected *L. delicatula* adult. Arrows indicate two of the stromata. (**b2**) Conidia. (**b3**) Colony on PDA at 28 d, obverse. (**b4**) Colony on PDA at 28 d, reverse. (c) *Cordyceps javanica*: (**c1**) Infected *L. delicatula* adult. (**c2**) Conidia. (**c3**) Colony on PDA at 37 d, obverse. (**c4**) Colony on PDA at 37 d, reverse. Cadaver scale bars: 3 mm. Conidial scale bars: 10 µm.

**Figure 3:**
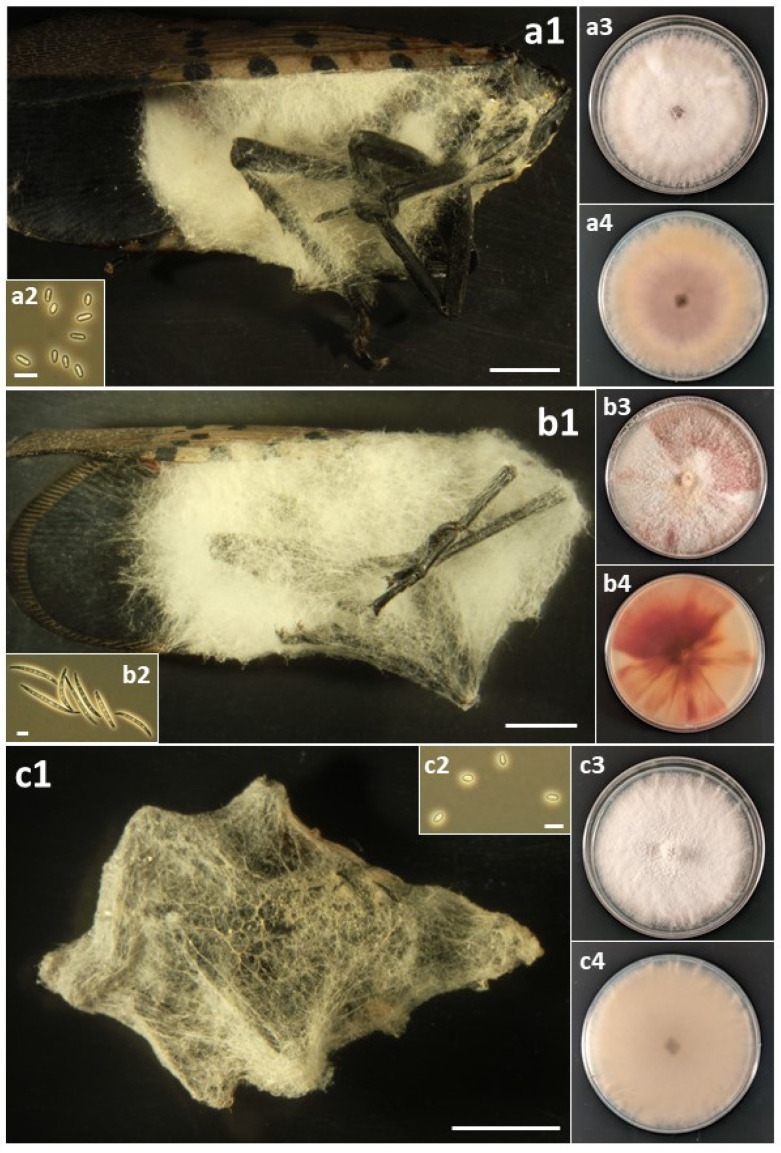
(a) *Flavocillium bifurcatum*. (**a1**) Infected *L. delicatula* adult. (**a2**) Conidia. (**a3**) Colony on PDA at 12 d, obverse. (**a4**) Colony on PDA at 12 d, reverse. (b) *Fusarium avenaceum*: (**b1**) Infected *L. delicatula* adult. (**b2**) Macroconidia. (**b3**) Colony on PDA at 10 d, obverse. (**b4**) Colony on PDA at 10 d, reverse. (c) *Fusarium concentricum*: (**c1**) Infected *L. delicatula* fourth-instar nymph. (**c2**) Microconidia. (**c3**) Colony on PDA at 12 d, obverse. (**c4**) Colony on PDA at 12 d, reverse. Cadaver scale bars: 3 mm. Conidial scale bars: 10 µm.

**Figure 4:**
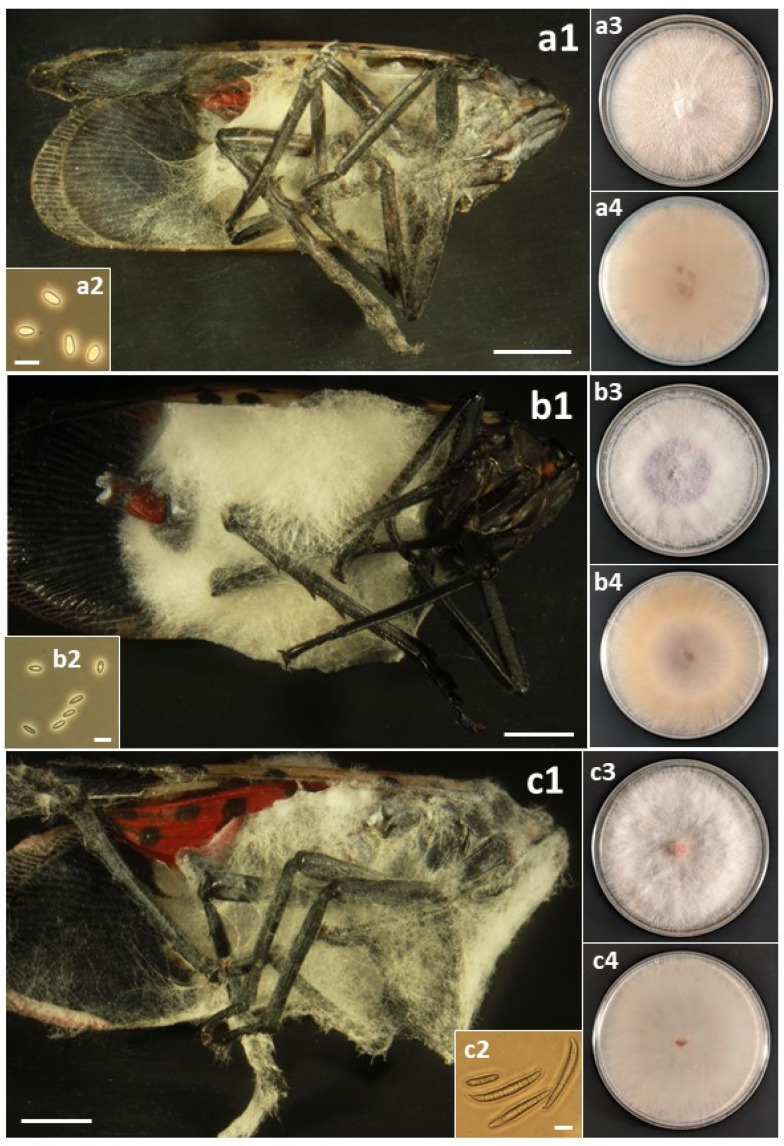
(a) *Fusarium falsibabinda*: (**a1**) Infected *L. delicatula* adult. (**a2**) Microconidia. (**a3**) Colony on PDA at 12 d, obverse. (**a4**) Colony on PDA at 12 d, reverse. (b) *Fusarium fujikuroi*: (**b1**) Infected *L. delicatula* adult. (**b2**) Microconidia. (**b3**) Colony on PDA at 11 d, obverse. (**b4**) Colony on PDA at 11 d, reverse. (c) *Fusarium graminearum*: (**c1**) Infected *L. delicatula* adult. (**c2**) Macroconidia. (**c3**) Colony on PDA at 10 d, obverse. (**c4**) Colony on PDA at 10 d, reverse. Cadaver scale bars: 3 mm. Conidial scale bars: 10 µm.

**Figure 5:**
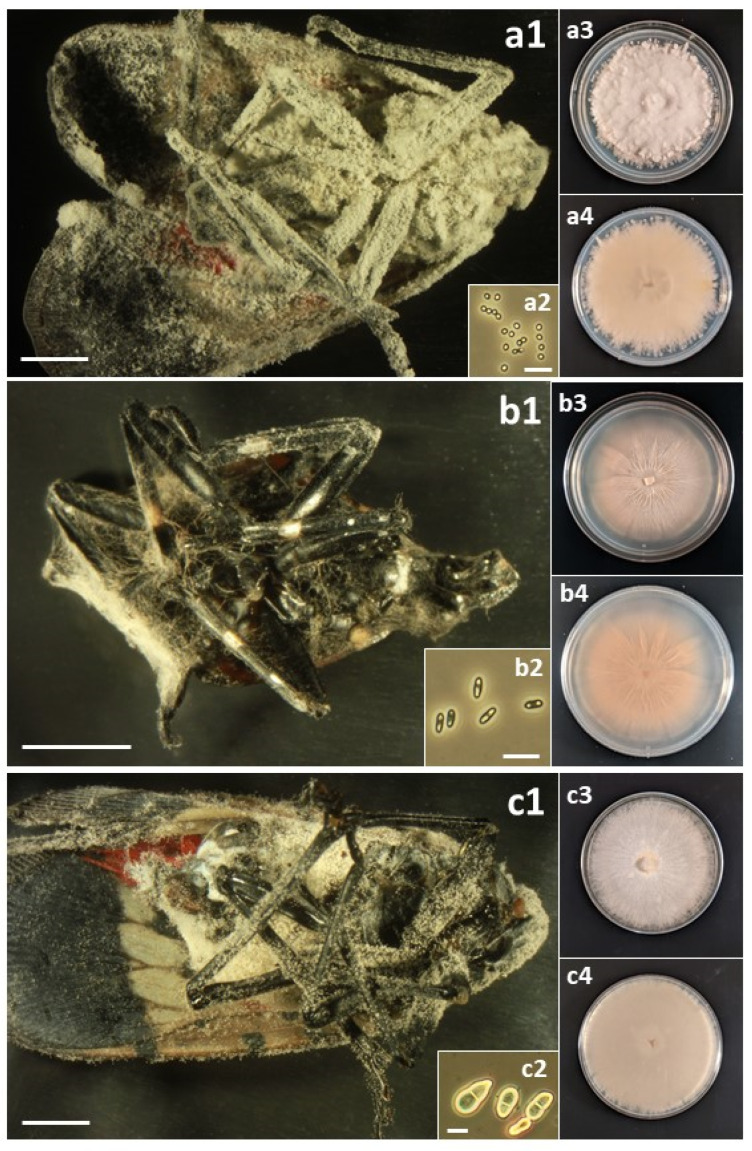
(a) *Samsoniella* sp.: (**a1**) Infected *L. delicatula* adult. (**a2**) Conidia. (**a3**) Colony on PDA at 48 d, obverse. (**a4**) Colony on PDA at 48 d, reverse. (b) *Sarocladium strictum*: (**b1**) Infected *L. delicatula* fourth-instar nymph. (**b2**) Conidia. (**b3**) Colony on PDA at 48 d, obverse. (**b4**) Colony on PDA at 48 d, reverse. (c) *Trichothecium roseum*: (**c1**) Infected *L. delicatula* adult. (**c2**) Conidia. (**c3**) Colony on PDA at 10 d, obverse. (**c4**) Colony on PDA at 10 d, reverse. Cadaver scale bars: 3 mm. Conidial scale bars: 10 µm.

A total of sixteen species in six families of the Hypocreales were isolated from field-collected *L. delicatula* (Table 3). For most species, fungal outgrowth from cadavers did not provide quick identification; in the field, there was no fungal outgrowth from living insects and rarely from cadavers, and only after collection and incubation under humid conditions did outgrowth occur. Then, for most opportunistic pathogens, white, cobweb-like outgrowth occurred (Figure 1, Figure 2, Figure 3, Figure 4 and Figure 5), and both microscopy and sequencing were required for identification. The exception was *B. bassiana*, for which cadavers were more readily recognizable with abundant bright white mycelium and dense balls of conidia (=‘spore balls’) [74]. Stromata grew from one cadaver of an *L. delicatula* adult killed by *C. cateniannulata* (Figure 2(b1)), but this growth occurred long after collection.

### 3.2. Pathogen Prevalence

Merging reared insects and collected cadavers in 2022, *B. bassiana* was collected most frequently (59.2% of all entomopathogenic fungi), while *F. fujikuroi* (16.8%) and *Sarocladium strictum* (12.3%) were the next most frequently found pathogens (Table 5). These three species were also the most widely distributed, being found at 5–6 of the 6 collection sites. Although we sampled throughout the season, the remaining species were rarely collected and were never found at all sites (Table 5). The most common genus was *Fusarium*, with five species collected, yet only *F. fujikuroi*-infected *L. delicatula* were collected more than five times.

**Figure 6:**
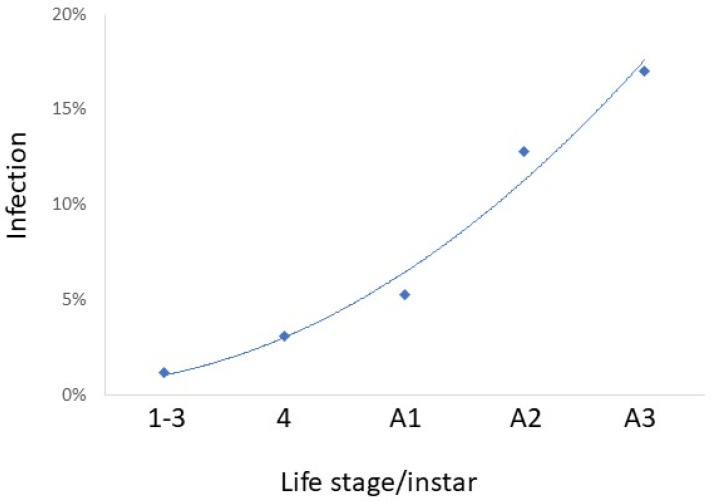
Percent *Lycorma delicatula* collected in the field that were infected with the 14 fungal pathogens collected in 2022 by life stage/instar. Field-collected living nymphs and adults were reared on potted *Ailanthus altissima* under quarantine for 14 d after collection or were collected in the field as cadavers of recently dead *L. delicatula*. All fungal species (Table 3) except *Cordyceps javanica* and *Colletotrichum fioriniae* were collected in 2022.

**Figure 7:**
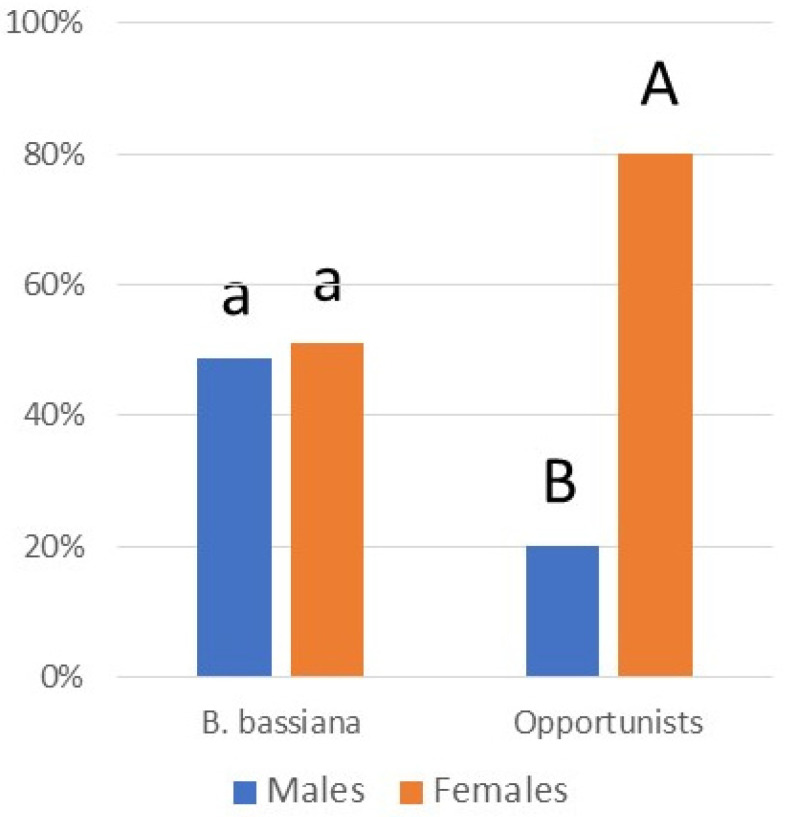
Male versus female adults infected by *B. bassiana* versus opportunistic pathogens in 2022. ‘Opportunistic pathogens’ killing adults included *A. muscarius*, *F. concentricum*, *F. falsibabinda*, *F. fujikuroi*, *F. graminearum*, and *S. strictum*. Letters above bars demonstrate statistical differences between numbers of males versus females infected, separately by pathogen type.

While infection prevalence by species was often low, together, these pathogens caused a total of 6.7% mortality. Considering all 14 pathogens collected in the field in 2022 (this includes all species in Table 3 except *C. javanica* and *C. fioriniae*), percentages of *L. delicatula* infection increased through the season, with little infection in nymphs and infection increasing especially through adult stages A2 and A3 (R^2^ = 0.9777) (Figure 6). Comparisons among instars/stages revealed that infection was equivalent for stages A2 and A3, when infection levels were greater than other instars/stages (chi-squared tests; *p* < 0.05). Among adults infected by opportunistic pathogens (i.e., all species except *B. bassiana*), significantly more adults females were infected than adult males (chi-squared = 21.600; *p* < 0.0001) (Figure 7). Among adults infected by *B. bassiana*, numbers of infected females were not significantly different from those of males (chi-squared = 0.047; *p* = 0.8292) (Figure 7).

In 2022, among cadavers of recently dead *L. delicatula*, infection by *B. bassiana* was much more abundant (81.7%) than *B. bassiana* infections among reared insects (18.7%) (chi-squared = 40.8427; *p* < 0.00001). Few cadavers of nymphs were found (Table 5). Also, few cadavers of the opportunistic pathogens were found at sites (18.8% of cadavers), and most of these pathogens were discovered through rearings (68.6% of entomopathogenic fungi from rearings).

## 4. Discussion

This study increased the known number of naturally occurring entomopathogenic fungi infecting *L. delicatula* from 4 species [24,27] to a total of 19. Numerous isolated fungal species that we report as pathogens of *L. delicatula* are not well known as entomopathogens or have not been reported as entomopathogens previously, e.g., *F. falsibabinda*. Our sampling demonstrated the highest levels of infection among mid- and late-stage adults (i.e., from September to November) (Figure 6). *Lycorma delicatula* adults generally live from late July to early November [75], which is a long life compared to that of adults of many insect species. This host is invasive, with no other members of the same family (Fulgoridae) in the areas sampled [76], so we assume that these pathogens are generalists and not specialists. Boomsma et al. [77] hypothesized that in the case of generalist entomopathogenic fungi, average spore doses usually present in the environment would be low and, as such, these possible insect pathogens would usually only be able to kill compromised hosts. Studies conducted with *B. bassiana* have shown that as *L. delicatula* adults age, they become weaker ([28]). As confirmation, we found the highest levels of infection in the A2 and A3 adult stages that had already been adults for at least one month. Because *L. delicatula* are invasives relatively new to the areas sampled, many of the pathogens being reported in this study could be considered opportunists that have adopted this invasive as a new food source.

In general, fungi have diverse ecological functions, including acting as saprophytes, endophytes, and pathogens of plants, animals, and fungi. Aside from *B. bassiana* [45], the pathogens identified in this study have not been reported previously as *L. delicatula* pathogens and have usually been reported as having additional ecological functions, leading to multifunctional lifestyles. For example, *C. fioriniae*, which killed *L. delicatula* in this study, is also well known as a plant endophyte and plant pathogen, and *F. avenaceum* and *F. graminearum* are well known as plant pathogens (see Table 3). This provides further evidence that many of the entomopathogenic fungi identified in this study are accidental opportunists that, in the space and time when they were collected, were acting as insect pathogens. In fact, secondary metabolites that are considered toxic to insects have been identified from fungi not principally known as insect pathogens; for example, many *Fusarium* species, including *Fusarium concentricum*, produce beauvericin [56], and *S. strictum* produces bassianolide, among other compounds [62].

Perhaps the occurrence and impact of these accidental opportunists are not recognized more frequently because their fungal outgrowth on cadavers is often not evident or easily identifiable. For example, several days after host death, bodies of *L. delicatula* killed by *B. bassiana* become covered with white mycelium, while many species in this study often became covered with paler, cobweb-like mycelium (Figure 1, Figure 2, Figure 3, Figure 4 and Figure 5). One significant exception was *C. cateniannulata* (Figure 2(b1)), from which large stromata grew. However, we only collected one specimen killed by *C. cateniannulata*, and cadavers killed by this fungus during pathogenicity tests did not produce stromata.

It is possible that our intensive sampling in 2022, when we sampled nymphs extensively as well as adults, could have led to underestimated infection levels compared with other years due to abiotic factors. During July and August 2022, there were drought conditions in the areas being sampled (Reading area and East Stroudsburg, Pennsylvania [78]), which would have decreased fungal transmission. It was surprising not to find *M. pemphigi* or *O. delicatula* [27] in 2022, especially as *M. pemphigi* had been collected in 2018 at one of the sites that were intensively sampled in 2022. *Batkoa major* had been very abundant in early October 2018 during a period with abundant rainfall and was recovered from field-collected *L. delicatula* during 2019–2021 (E.H.C. and D.C. Harris, unpublished data). However, during 2022, under intensive sampling but drier conditions, *B. major* infections were not observed.

Although the low levels of infection by most of these species will not individually have a strong impact on population suppression, when added together, the impact is greater. Most infections were recorded in stages A2 and A3 (R^2^ = 0.9777) (Figure 6), when mating and oviposition occur [73]. Among infected A2- and A3-stage adults, equal numbers of females and males were infected with *B. bassiana*, but among the opportunistic species, far more females were infected than males (Figure 7). Further studies are necessary to quantify the impact of infection by these fungi on *L. delicatula* population densities under field conditions.

Why would so much more infection occur among adults than nymphs? First, adults begin to eclose in July, and many live until early November [75]. Each nymphal instar persists for approximately 2 weeks [73], while individual adults can live >4 months [75]. So, adults are present in the environment much longer than nymphs. The soil is generally considered a reservoir for spores of hypocrealean entomopathogenic fungi [79]. At the sites sampled, nymphs were rarely recovered near the forest floor. In contrast, winged adults are highly mobile and often feed on tree roots or just above the base of the tree, close to the soil (unpubl. data). We hypothesize that by feeding in these locations, adults are more likely to become infected compared with nymphs.

*Fusarium falsibabinda* has not been reported previously as an insect pathogen. In this case, our laboratory isolated and assessed Koch’s postulates for this species by random chance. During this study, many more fungal species, including more *Fusarium* species, were isolated from dead *L. delicatula* in 2022, but we prioritized testing species known to be insect pathogens. Worldwide, several dozen *Fusarium* species have been reported as insect pathogens, but all of these are generally known from few isolates and host species [56,80]. In some reported instances, *Fusarium* species were only isolated from a challenged insect with the ‘*Galleria* bait method’ [81], and Koch’s postulates were confirmed using lab colonies of *Galleria mellonella* instead of wild insects. Epizootics in insect populations caused by *Fusarium* species have been documented, but not due to any of the *Fusarium* species isolated in this study [82]. However, the white, cobweb-like growth on cadavers exhibited by *Fusarium* species (Figure 3(b1,c1) and Figure 4(a1,b1,c1)) is shared with the other opportunistic species in this study, making species identifications in the field difficult and likely contributing to an observation bias against species of *Fusarium* and hypocrealean genera other than the more easily recognizable *B. bassiana*.

Numerous opportunistic pathogens we have reported are less well known. This is the first report of *C. cateniannulata*, *Clonostachys eriocamporesii*, *F. bifurcatum*, *F. falsibabinda*, and *Samsoniella* sp. in North America. However, North American isolates of some of these taxa may have been previously reported under other names. This is the first study to conduct Koch’s postulates with a species of *Samsoniella* against insects, although species of *Samsoniella* have been isolated from dead insects [83] and have also been found to be pathogens of nematodes [84]. Future taxonomic work is needed to determine which species of this recently named genus are present in North America.

*Cladosporium* and *Mucor* species have recently been confirmed as important pathogens in hemipteran populations, with potential for biological control [85,86,87]. Unfortunately, during this study, we were unable to evaluate the pathogenicity of additional potentially pathogenic species such as these that that were isolated from dead *L. delicatula*. Although in 2022, fungi were isolated from dead *L. delicatula*, the cadavers had not been surface-sterilized, so tests of pathogenicity were needed to confirm pathogenicity. Because we emphasized species already known to be entomopathogens, this study perhaps underestimated opportunists present in the field that were infecting *L. delicatula*, and future trials using Koch’s postulates could help to identify additional entomopathogenic species.

Although in this study we report 15 species that were previously not known as pathogens of *L. delicatula*, we hypothesize that 2021–2023 were probably not the first years for the infection of these invasives by these opportunistic generalists. *Lycorma delicatula* was first found in only one county in Pennsylvania in 2014 [21] and has been spreading since [20]. During our earlier studies [24,27], we were limited in methodology, and these opportunists could have gone undetected or could have been removed from the environment by scavengers, saprophytes, or weathering, preventing collection. As *L. delicatula* is constantly spreading, further studies could discover more species of entomopathogens infecting this invasive in new areas.

## Figures and Tables

**Table 1:** Sites for sampling *Lycorma delicatula* in Pennsylvania and New York, 2021–2023.

Site	County	State	GPS	Year(s) Sampled
Angora Fruit Farm, Lower Alsace Twp.	Berks	Pennsylvania	40.35846 N, 75.88323 W	2021, 2022, 2023
Penn Ave., Sinking Spring, Lower Heidelberg Twp.	Berks	Pennsylvania	40.32700 N, 76.04683 W	2022, 2023
Glen Run Nature Preserve, Stroud Twp.	Monroe	Pennsylvania	40.96950 N, 75.19004 W	2021, 2022, 2023
Minisink Park, Delaware Water Gap	Monroe	Pennsylvania	40.98622 N, 75.13695 W	2022
Cherry Valley Rd., Stroud Twp.	Monroe	Pennsylvania	40.97055 N, 75.17941 W	2022
Boys & Girls Club, Main St., Owego	Tioga	New York	42.11008 N, 76.25717 W	2023

**Table 2:** Primers used for PCR and sequencing.

Locus	Primer	Primer Sequence	Reference
ITS	ITS1	TCCGTAGGTGAACCTGCGG	White et al., 1990 [29]
	ITS1 mod	TCCGTAGGTGAACCTTGCGG	Korabečná et al., 2003 [38]
	ITS4	TCCTCCGCTTATTGATATGC	White et al., 1990 [29]
LSU	LR5	ATCCTGAGGGAAACTTC	Vilgalys and Hester, 1990 [30]
	LR0R ^1^	ACCCGCTGAACTTAAGC	Lutzoni, 2014 [39]
RPB1	CRPB1	CCWGGYTTYATCAAGAARGT	Castlebury et al., 2004 [31]
	RPB1-Cr	CNGCDATNTCRTTRTCCATRTA	Matheny et al., 2002 [40]
RPB2	RPB2-5F	GAYGAYMGWGATCAYTTYGG	Liu et al., 1999 [34]
	RPB2-5F2	GGGGWGAYCAGAAGAAGGC	Sung et al., 2007 [32]
	RPB2-7cR	CCCATRGCTTGYTTRCCCAT	Liu et al., 1999 [34]
TEF1-α	EF-1	ATGGGTAAGGARGACAAGAC	O’Donnell et al., 1998 [41]
	EF-2	GGARGTACCAGTSATCATGTT	O’Donnell et al., 1998 [41]
	983F	GCYCCYGGHCAYCGTGAYTTYAT	Rehner and Buckley, 2005 [35]
	2218R	ATGACACCRACRGCRACRGTYTG	Rehner and Buckley, 2005 [35]
TUB2	T1	AACATGCGTGAGATTGTAAGT	O’Donnell and Cigelnik, 1997 [42]
	T2	TAGTGACCCTTGGCCCAGTTG	O’Donnell and Cigelnik, 1997 [42]

^1^ Referenced as (Vilgalys, unpublished).

**Table 3:** Hypocrealean pathogens isolated from *Lycorma delicatula* (White, 1845).

Kingdom Fungi	Functional Roles ^1^	References for Roles
Division Ascomycota		
Subdivision Pezizomycotina		
Class Sordariomycetes		
Order Hypocreales		
**Family Cordycipitaceae**		
*Akanthomyces muscarius* (Petch) Spatafora, Kepler and B. Shrestha, 2017	EPF, MP	Saidi et al., 2023 [43]; Broumandnia and Rajabpour, 2021 [44]
*Beauveria bassiana* (Bals.-Criv.) Vuill., 1912	EPF, PEP	Clifton et al., 2019, 2023 [24,45]; Hajek and Meyling, 2018 [46]
*Cordyceps cateniannulata* (Z.Q. Liang) Kepler, B. Shrestha, and Spatafora, 2017	EPF	Montes-Bazurto et al., 2020 [47]
*Cordyceps javanica* (Bally) Kepler, B. Shrestha, and Spatafora, 2017	EPF	Clifton and Hajek, 2021 [28]
*Flavocillium bifurcatum* H. Yu, Y.B. Wang, Y. Wang, Q. Fan, and Zhu L. Yang, 2020	EPF	Wang et al., 2020 [48]
*Samsoniella* sp.	EPF	This study
**Family Bionectriaceae**		
*Clonostachys eriocamporesii* R.H. Perera and K.D. Hyde, 2020	EPF	Rodrigues et al., 2022 [49]
*Clonostachys rosea* (Link) Schroers, Samuels, Seifert, and W. Gams, 1999	EPF, MP	Yang et al., 2021 [50]; Sun et al., 2020 [51]
**Family Nectriaceae**		
*Fusarium avenaceum* (Fr.) Sacc., 1886	EPF, PP, PEP	Batta, 2012 [52]; Rajagopal and Suryanarayanan, 2000 [53]; Uhlig et al., 2007 [54]
*Fusarium concentricum* Nirenberg and O’Donnell, 1998	EPF, PP	Qiu et al., 2023 [55]
*Fusarium falsibabinda* M.M. Wang and L. Cai, 2022	EPF	This study
*Fusarium fujikuroi* Nirenberg, 1976	EPF, PP	Sharma et al., 2018 [56]; Dewing et al., 2022 [57]
*Fusarium graminearum* Schwabe, 1839	EPF, PP, PEP	Ameen, 2012 [58]; Trail, 2009 [59]; Lofgren et al., 2018 [60]
**Family Sarocladiaceae**		
*Sarocladium strictum* (W. Gams) Summerb., 2011	EPF, PP, PEP, MP	El-Sayed et al., 2020 [61]; Blaszczyk et al., 2021 [62]; Tagne et al., 2002 [63]; Racedo et al., 2013 [64]; Kim 2002 [65]; This study
**Family Glomerellaceae**		
*Colletotrichum fioriniae* (Marcelino and Gouli) Pennycook, 2017	EPF, PP, PEP	Gonzalez et al., 2023 [66]; Marcelino et al., 2009 [67,68]
**Family Myrotheciomycetaceae**		
*Trichothecium roseum* (Pers.) Link, 1809	EPF, PP, MP	Batta, 2020 [69]; Götz and Karbowy-Thongbai, 2023 [70]; Zhu et al., 2022a [71]

^1^ EPF = entomopathogenic fungi; PP = plant pathogen; PEP = plant endophyte; MP = mycoparasite. ‘This study’ indicates that this is the first report of this pathogen being an EPF.

**Table 4:** Voucher and GenBank accession numbers for exemplar isolates of entomopathogenic species collected and identified in Pennsylvania and New York, from 2021–2023 ^1^.

Fungal Species	ARSEF Number	ITS	LSU	RPB1	RPB2	TEF1-α	TUB2
*Akanthomyces muscarius*	14661	OR577160 ^2^	OR575222	OR593718	OR593721	OR593725	
*Clonostachys eriocamporesii*	14696	OR582991 ^2^				OR602799	
*Clonostachys rosea*	14682	OR577161				OR593726	
*Colletotrichum fioriniae*	14695	OR583017					OR672136
*Cordyceps cateniannulata*	14662	OR577162 ^2^	OR575223	OR593719		OR593727	
*Cordyceps javanica*	14690	OR577169					OR672137
*Flavocillium bifurcatum*	14694	OR582994 ^2^	OR577040	OR602848	OR602852	OR602802	
*Fusarium avenaceum*	14691	OR577163				OR593728	
*Fusarium concentricum*	14687	OR652385				OR593729	
*Fusarium falsibabinda*	14667	OR577164 ^2^				OR593730	
*Fusarium fujikuroi*	14677	OR577165 ^2^			OR593722	OR593731	
*Fusarium graminearum*	14692	OR577166 ^2^			OR593723	OR593732	
*Samsoniella* sp.	14689	OR577167		OR593720	OR593724	OR672131	OR672138
*Sarocladium strictum*	14693	OR577168 ^2^				OR672132	
*Trichothecium roseum*	14697	OR583015 ^2^				OR672134	

^1^ *B. bassiana* is not included in this table due to the extensive pre-existing analyses of *B. bassiana* biodiversity in Pennsylvania [45]. ^2^ Isolates that were amplified and sequenced with ITS1 (mod) (Table 2) for the forward primer.

**Table 5:** Distribution of 16 entomopathogenic fungal species across sites and life stages, isolated from *Lycorma delicatula* in 2021–2023.

Fungal Species	# Sites with Fungus	Instars Infected							
		1	2	3	4	A1	A2	A3	Total by Row
*Akanthomyces muscarius*	3				2	1	1	5	**9**
*Beauveria bassiana*	5			1	1	4	32	50	**88**
*Clonostachys eriocamporesii*	1				1				**1**
*Clonostachys rosea*	2		1		2				**3**
*Colletotrichum fioriniae*	1			1					**1**
*Cordyceps cateniannulata*	1							1	**1**
*Cordyceps javanica*	1				2				**2**
*Flavocillium bifurcatum*	1			1					**1**
*Fusarium avenaceum*	4		2	1	1				**4**
*Fusarium concentricum*	3	1				2	1		**4**
*Fusarium falsibabinda*	2						1	4	**5**
*Fusarium fujikuroi*	6			1	6	14	9		**30**
*Fusarium graminearum*	3						3	2	**5**
*Samsoniella* sp.	1				1				**1**
*Sarocladium strictum*	5				3	6	10	3	**22**
*Trichothecium roseum*	1				2				**2**

## Data Availability

The data supporting the findings of study are available upon reasonable request to the corresponding author.

## Supplementary Materials

The following supporting information can be downloaded at: https://www.mdpi.com/2075-4450/14/12/912/s1: Table S1: GenBank accession numbers for additional sequenced isolates of enotomopathogenic fungi infecting *L. delicatula*; Table S2: Methods used for challenging *L. delicatula* to confirm pathogenicity of fungal species.
