# Supplementary material for: Accumulation of Fungal Pathogens Infecting the Invasive Spotted Lanternfly, Lycorma delicatula
Source: Insects. 2023 Nov 27;14(12):912. doi: 10.3390/insects14120912 (PMC10871119; doi:10.3390/insects14120912)
Supplement: Supplementary file 1 [file insects-14-00912-s001.zip › insects-2718669-supplementary.pdf]

Table S1. GenBank accession numbers for additional sequenced isolates of enotomopathogenic fungi infecting *L. delicatula*.

**Fungal species**	**Isolate**	**ITS**	**LSU**	**RPB1**	**RPB2**	**TEF 1-α**
Akanthomyces muscarius	27-Jul-AFF4a	OR582987	OR577036	OR602845		OR602796
Akanthomyces muscarius	27-Jul-AFF5a	OR582988	OR577037			OR672128
Akanthomyces muscarius	2-Nov-GR29b	OR582989 [1]	OR577038	OR602846	OR602850	OR602797
Akanthomyces muscarius	7-Sep-AFF-FC10	OR582990 [1]	OR577039	OR602847	OR602851	OR602798
Akanthomyces muscarius	23-Sep-GR-FC1	OR582986		OR602844		OR672129
Akanthomyces muscarius	12-Oct-AFF-FC5	OR582985 [1]	OR577035	OR602843	OR602849	OR602795
Clonostachys rosea	20-Jul-GR7a	OR582992 [1]				OR602800
Clonostachys rosea	27-Jul-GR5a	OR582993 [1]				OR602801
Cordyceps javanica	SS-L3-14F	OR583014				OR672130
Fusarium avenaceum	22-Jun-CCC2b	OR582997 [1]				OR602805
Fusarium avenaceum	13-Jul-F2	OR582995 [1]				OR602803
Fusarium avenaceum	20-Jul-AFF4	OR582996				OR602804
Fusarium concentricum	8-Jun-F1					OR602808
Fusarium concentricum	3-Aug-AFF7					OR602807
Fusarium concentricum	23-Sep-CCC10	OR582998				OR602806
Fusarium falsibabinda	5-Oct-GR18					OR602812
Fusarium falsibabinda	2-Nov-F23	OR582999 [1]				OR602809
Fusarium falsibabinda	2-Nov-F31	OR583000				OR602810
Fusarium falsibabinda	2-Nov-GR-FC16	OR583001				OR602811
Fusarium fujikuroi	13-Jul-F1				OR602853	OR602816
Fusarium fujikuroi	27-Jul-AFF9a	OR583004			OR602854	OR602824
Fusarium fujikuroi	27-Jul-F1				OR602855	OR602826
Fusarium fujikuroi	3-Aug-AFF4					OR602828
Fusarium fujikuroi	3-Aug-GR12					OR602829
Fusarium fujikuroi	10-Aug-GR2					OR602814
Fusarium fujikuroi	10-Aug-GR9					OR602815

Fusarium fujikuroi	10-Aug-GR12			OR602813
Fusarium fujikuroi	24-Aug-AFF3	OR583003		OR602819
Fusarium fujikuroi	24-Aug-GR2			OR602821
Fusarium fujikuroi	24-Aug-GR15			OR602820
Fusarium fujikuroi	24-Aug-GR22			OR602822
Fusarium fujikuroi	24-Aug-GR25			OR602823
Fusarium fujikuroi	7-Sep-GR7			OR602837
Fusarium fujikuroi	7-Sep-GR22	OR583008		OR602836
Fusarium fujikuroi	7-Sep-CCC1			OR602833
Fusarium fujikuroi	7-Sep-CCC13			OR602834
Fusarium fujikuroi	7-Sep-CCC15W	OR583007		OR602835
Fusarium fujikuroi	23-Sep-GR27			OR602818
Fusarium fujikuroi	23-Sep-F24	OR583002		OR602817
Fusarium fujikuroi	5-Oct-GR10	OR583006		OR602830
Fusarium fujikuroi	5-Oct-GR15			OR602831
Fusarium fujikuroi	5-Oct-GR16			OR602832
Fusarium fujikuroi	O-L4-2	OR583018		OR602842
Fusarium fujikuroi	27-Jul-AFF-FC3			OR602825
Fusarium fujikuroi	29-Sep-AFF-FC19Y	OR583005		OR602827
Fusarium graminearum	5-Oct-AFF31	OR583011 [1]	OR602857	OR602840
Fusarium graminearum	5-Oct-AFF33	OR583012 [1]	OR602858	OR602841
Fusarium graminearum	2-Nov-GR15	OR583010	OR602856	OR602839
Fusarium graminearum	2-Nov-CCC22	OR583009		OR602838
Sarocladium strictum	27-Jul-F3	OR583013 [1]		OR672133
Trichothecium roseum	AFF-L3-4C	OR583016		OR672135

[1] Isolates which were amplified and sequenced with ITS1 (mod) for the forward primer.

Table S2. Methods used for challenging *L. delicatula* to confirm pathogenicity of fungal species.

**Fungal species**	**Conditions promoting conidial production**				**Conidial concentration (conidia/ml)**	**Instars tested**	**Testing results**[1]
	**Media**	**Parafilm**	**Light**	**Temperature (°C)**			
Akanthomyces muscarius	PDA	Yes	Dark	23	6.7×10^7	A3	Visual ID
Clonostachys eriocamporesii	1/3 PDA	No	12:12 light	28	1×10^7	A2	KP
Clonostachys rosea	PDA	Yes	Dark	24	1×10^7	1–2	KP
Colletotrichum fioriniae	PDA	Yes	Dark	24	1×10^7	A2	KP
Cordyceps cateniannulata	PDA	Yes	Dark	26	7.4×10^6	A3	Visual ID
Cordyceps javanica	PDA	Yes	Dark	24	1×10^7	A3	KP
Flavocillium bifurcatum	PDA	Yes	Dark	24	1×10^7	A2	KP
Fusarium avenaceum	PDA	No	12:12 UV	24	1×10^7	A1	KP
Fusarium concentricum	PDA	Yes	Dark	24	1×10^7	4	KP
Fusarium falsibabinda	PDA	Yes	Dark	23	3.5×10^7	A3	Visual ID
Fusarium fujikuroi	PDA	Yes	Dark	24	1×10^7	2–3	KP
Fusarium graminearum	PDA	No	12:12 UV	24	1×10^7	A1	KP
Samsoniella sp.	PDA	Yes	Dark	24	1×10^7	4	KP
Sarocladium strictum	PDA	Yes	Dark	24	1×10^7	4	KP
Trichothecium roseum	SDA	Yes	Dark	24	1×10^7	A2	KP

[1] KP = Koch's postulates (reisolation from cadaver and ID); Visual ID = in 2021, we did not reisolate from infected insects but visually checked fungal outgrowth and conidia from cadavers.
